# Patient Centeredness in Orthognathic Surgery

**DOI:** 10.3390/clinpract11010014

**Published:** 2021-02-05

**Authors:** Alexandre R. Vieira, Maria C. O. Prinz

**Affiliations:** 412 Salk Pavilion, Department of Oral Biology & Craniofacial Sciences, School of Dental Medicine, University of Pittsburgh, 335 Sutherland Drive, Pittsburgh, PA 15261, USA; ma_prinz@hotmail.com

**Keywords:** orofacial pain, orthognathic surgery, patient centeredness, temporomandibular joint dysfunction, informed consent

## Abstract

Patient centeredness in planning treatment and research has become paramount. The goal of this report was to describe a complex case in which untreated chronic pain was not properly addressed to reflect on the need to establish alternative protocols for controlling chronic orofacial pain. When a female underwent orthognathic surgery to correct her occlusion, she not only ended up with a worse occlusion, she developed chronic orofacial pain that could not be treated by opioids and only improved after the use of neuropathic medication, and finally disappeared after the use of low-level laser therapy. There is a need to incorporate alternative nonpharmacological approaches to manage chronic pain. Further, what the patient’s goals are for their treatments should be given priority in case of elective procedures.

## 1. Introduction

Pain that lasts for at least three to six months is defined as chronic and originates from many sources. It appears to be a problem that a growing number of individuals today have experienced one way or the other. Chronic pain impacts daily life and mental health.

The world is experiencing an unprecedented crisis in the control of pain, including chronic pain. Whereas a few countries, including the United States, Canada, and Australia, use too much opioids, the rest of the globe includes millions of people who have untreated pain [1] In Nigeria, only 0.2% of the need for pain palliative care is met, Mexico meets 36% of palliative care need, and Canada has 3.090% of the need of palliative care available for distribution [2]. On the one hand, there is a need to create mechanisms for low-income countries to have access to proper amounts of pain relief medicine. On the other hand, alternative approaches to control, reduce, or eliminate chronic pain are needed to address the crisis of over-prescription of opioids in North America and Australia.

In respect to pain specifically in the face, there is evidence that orthognathic surgery may provide relief for individuals with pre-existing signs and symptoms of orofacial pain or temporomandibular joint dysfunction (TMD) [3]. Results are mostly predictable, but complications after surgery are common enough to be of concern. TMD, when present prior to surgery, may be reduced in certain cases. When retrognathic patients underwent bilateral sagittal split osteotomy to advance the mandible, they were 41% more likely to have their TMD symptoms improved. This effect was not seen when Le Fort I osteotomy was also performed. In the case of prognathic cases, overall there was also a reduction in TMD symptoms (close to 36%), and these results tended to be promising, independent from the setback of the mandible being achieved by bilateral sagittal split osteotomy, intraoral vertical ramus osteotomy, Le Fort I osteotomy, or combinations thereof [4]. What this kind of analysis cannot reconcile is that some individuals with pre-surgery TMD symptoms improve, whereas some stay the same and some worsen. Similarly, some patients who had no symptoms prior to surgery may develop orofacial pain and/or TMD.

A noninvasive approach, low-level laser therapy, has been suggested for the treatment of postmastectomy lymphedema [5,6], alleviating sports injuries [7], and reducing the painful symptoms of oral mucositis [8,9,10,11,12,13,14,15,16]. However, low-level laser therapy is not widely used in the treatment of pain in general.

Orthognathic surgeries are elective, but since surgery in general has become much safer, it has been indicated for improving esthetics and function, without necessarily any prior painful symptoms. There are reports that the number of orthognathic surgeries overall has increased [17], lesser so when health insurance issues are involved [18,19]. Naturally, with more cases being operated, the number of complications and undesirable results are likely to increase. For those cases, we are yet to develop protocols and best practices on how to minimize sequalae. It is suggested that the identification of the type of mandibular retrusion of Class II individuals, using more sophisticated definitions (dimensional, rotational, positional, and any combinations of these three groups), may provide additional insight on how to better treat these cases [20]. It is apparent that early intervention with orthodontic functional appliances for children and adolescents with either unilateral of bilateral moderate to severe temporomandibular joint involvement and underlying juvenile idiopathic arthritis is beneficial to prevent more severe deviations of growth [21], and careful evaluation of young patients and indication of less invasive treatments minimize the need of later surgery.

The goal of this report is to reflect on a complex case that developed chronic pain after orthognathic surgery and was finally managed by low-level laser therapy as an alternative for the use of opioids, to discuss a number of aspects related to care of patients with chronic pain, including the concept of “patient centeredness”.

## 2. Orthognathic Surgery Indicated to Improve Jaw Function

**Figure 1:**
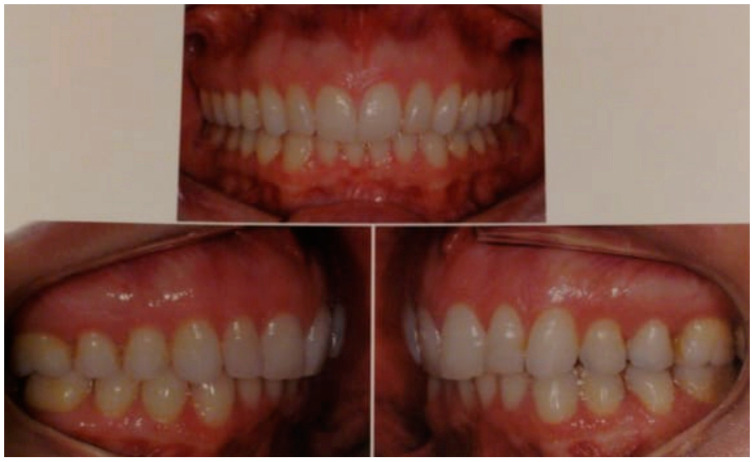
Preoperatory status of the occlusion. Orthognathic surgery was indicated to reduce the overjet and perceived discrepancy on the right side.

**Figure 2:**
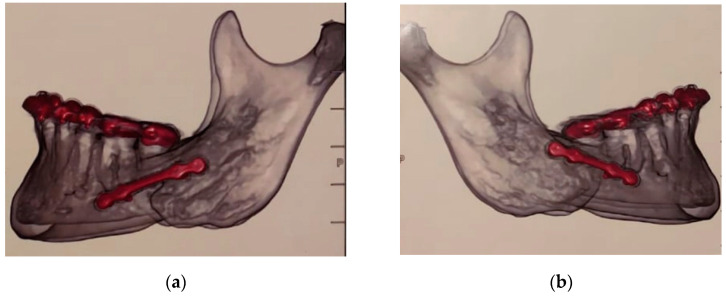
One-millimeter backward repositioning of the right side of the mandible (**a**) at the same time that the left side was advanced 1.5 mm (**b**).

A 37-yeal-old White female sought care for malocclusion due to a perceived difference in the mandible, according to her appearing to be slightly smaller on the right side (Figure 1). She had history of orthodontic treatment at a younger age. She received the suggestion that orthognathic surgery was the best indication due to the perception that orthodontic treatment alone would not be able to further correct her occlusion, therefore no pre- or postoperative orthodontic treatment was planned. There was also no record that an evaluation of the occlusion with an instrumentally guided analysis in an articulator was done. She was submitted to treatment by a maxillofacial surgeon without being presented with any major risks that entail a surgical procedure of this nature. She was made aware only of risks such as paresthesia, edema, restricted diet for a few weeks, and need to rest. She was concerned with any change in her physical profile since her only goal was to improve her bite. According to the portion of the medical record we had access to, the surgical plan outlined by her surgeon included a combined vertical movement of the maxilla (4 mm upward) with a rotation of the mandible after vertical bilateral osteotomy to allow a one-millimeter backward repositioning of the right side of the mandible at the same time that the left side was advanced 1.5 mm. This is puzzling since the records (Figure 2) showed that the classical Obwegeser (bilateral sagittal split osteotomy) was executed. We do not have information if presurgical guides were used with condyles positioned in a centric relationship or if the condyles were fixed before mandibular osteotomy. We also cannot tell why a bilateral sagittal osteotomy was not performed.

**Figure 3:**
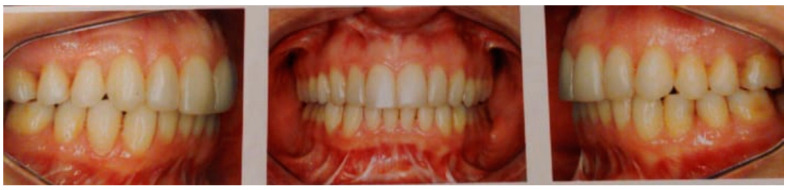
Postsurgical images show neglectable changes in the occlusion that, however, were accompanied by constant orofacial pain on the left side.

After initial recovery, the patient was showing signs of paresthesia and strong pain in the face on the left side that on a scale from 0 to 10 ranged from 6 to 8. She was allergic to a number of analgesic and anti-inflammatory drugs and therefore was never prescribed opioids. Aside from those adverse events, the patient was not satisfied with the changes in her facial profile and the fact of the perception of her occlusion to have worsen, and that a facial asymmetry was formed with complete loss of contact on the right side (Figure 3). This result is difficult to reconcile. A number of factors could have played a role, from the experience of the surgeon, to correct use or manufacturing of the surgical bite guide used. It is impossible to determine if any of these factors played a role, but for a surgical procedure that was indicated for such a small apparent change in the position of the jaws, a small mistake in any of the steps in preparation to and during the surgery could have led to the negative outcome described here.

**Figure 4:**
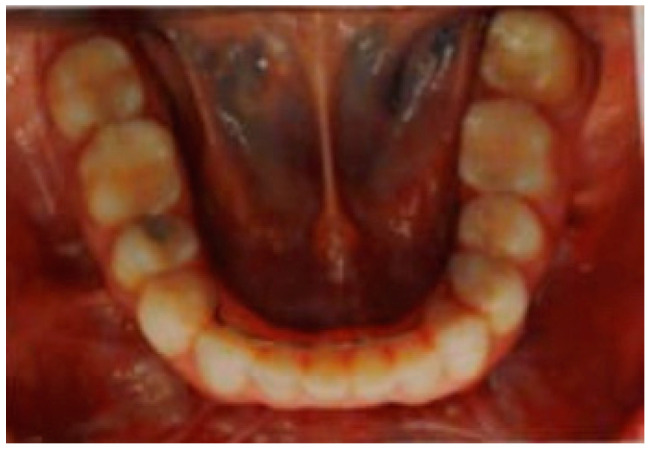
The second left mandibular molar received a root canal treatment despite being vital and some fibrotic scar tissue on the buccal aspect of the first left mandibular molar is noticeable.

After not having her orofacial pain addressed by the surgeon that she was under care of, the patient visited more than a dozen professionals in the following three years. There was no evidence that any screws were directly put on the mandibular canal, there was no suggestion for removing the plates, there were no obvious signs of discomfort originating from the temporomandibular joint, she was submitted to an endodontic treatment of her vital second left mandibular molar (Figure 4), and was finally prescribed amitriptyline, an antidepressant that is used for neuropathic pain that she used for three months. We cannot tell why the endodontic treatment was suggested and if sensitivity or vitality tests of the tooth were performed. The medication did finally reduce her facial pain from the 6 to 8 range to a 1 to 3 range. She decided to discontinue the use of the medication due to its side effects; however, the pain did not increase to the levels before the medication use, although the pain never “went away.” The numbness caused by the paresthesia became more noticeable once the pain subsided slightly, and facial sensitivity never returned to the patient.

**Figure 5:**
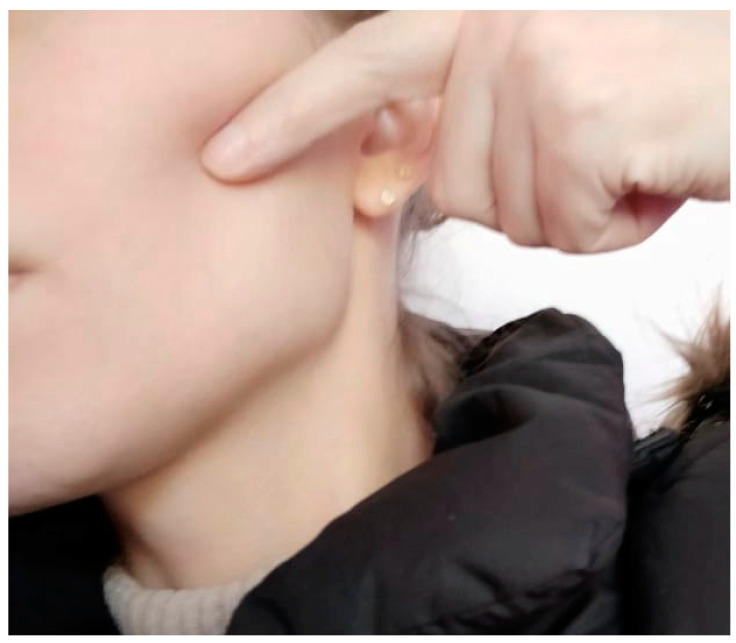
Site where the patient localized the origin of her facial pain.

Finally, after almost three years after the surgery and constant pain, the patient received the indication for a one-time treatment with low-level laser therapy. She received four applications of 40 s, each with a wavelength of 808 nm, power of 100 mW, for a total of 4 joules per application. Three applications were in the face, at the site where the patient identified her discomfort (Figure 5), then 1.5 mm anteriorly and 1.5 mm posteriorly of this point. The fourth application was intraoral, at the level of the buccal aspect of her first left mandibular molar, which she also identified as painful (Figure 4). The patient mentioned immediate relief of her pain that continued for the days and weeks that followed. However, according to her perception, the paresthesia became more apparent and a constant reminder of her ordeal. An evaluation after one year of the low-level laser therapy showed that the pain became only occasional and the intensity much milder than before.

## 3. Discussion

According to the Institute of Medicine, providing care that is respectful of, and responsive to, individual preferences, needs and values, and ensuring that patient values guide all clinical decisions is a fundamental need to current medical practice. In an extreme example, the use of a tattoo saying “do not resuscitate” to express end-of-life wishes when the person is incapacitated, although caused confusion at first, was corroborated by written records and ultimately respected [22]. Therefore, “mortality” may not be the outcome of most interest to patients, but rather a life without pain. A more contemporary viewpoint is incorporating outcome measures that are also relevant to patients along with outcomes that may be of specific interest to surgeons, care providers, and scientists [23].

The case presented here is an excellent example of how the patient’s wishes did not guide the planning and therefore the results of surgical treatment. A request to improve function due to an inadequate occlusion resulted in complete lack of occlusion on the left side, paresthesia, orofacial pain not originated from the temporomandibular joint, and facial asymmetry with additional changes in profile that caused disconcert. After three years and innumerous professional consultations that included an endodontic (root canal) treatment of a vital tooth, hours of personal time, and substantial financial burden, the patient still did not have her concern addressed. This is a clear example on how disconnected the professional care offered was to the patient wishes. We recommend that treatments be designed having in mind patient-centered outcomes. A reasonable protocol for orthognathic surgery may suggest the following conventional course of examination/therapy, which would have been applied to the patient presented here:

1. postoperatory 3-D imaging shortly after surgery to prove that the screws have not been inserted into the nerve canal;
2. either prosthodontic, but most preferably orthodontic measures, to achieve correct and stable occlusion. If prosthodontic treatment is considered, an instrumentally assisted analysis of the bite is necessary;
3. in case of persisting pain, removal of the plates after about 6–8 months post-surgery, since they can definitely contribute to painful sensations;
4. pain relieving medication/low-level laser application.

Since steps 1–3 were never implemented, and conventional pain medication was given directly without having found the underlying reason, we proposed a conservative treatment, which at that point was the only aspect of treatment options that were acceptable for the patient.

The mechanism on how low-level laser therapy at low doses operates to relieve pain likely include the enhancement of cell proliferation of fibroblasts [24,25,26,27], keratinocytes [28], endothelial cells [29], and lymphocytes [30,31]. Proliferation is probably the result of photostimulation of the mitochondria, leading to neovascularization, angiogenesis, and increase of collagen synthesis (through activation of transcription) to aid in the healing of acute and chronic wounds [24,32,33,34,35,36,37,38,39]. In the present case, pain was localized on the left side and after three years, facial muscles lost tonicity. The patient responded well to amitriptyline, an antidepressant that is used for neuropathic pain, and her pain, which used to fluctuate between 6 and 8 on a scale of 0 to 10, was less intense, fluctuating between 1 and 3. The application of low-level laser in this case likely led to an immediate decrease in mitochondrial membrane potential, leading to a nociceptor blockade, an increase in serotonin and endorphin levels (since the patient previously responded well to neuropathic medication) [40,41,42,43,44,45].

We strongly believe that low-level laser therapy should be more broadly used, in particular for cases of orofacial pain involving, or not, the temporomandibular joint. Women suffer from orofacial pain at least twice as much than men [46], and there is a general perception that women issues are understudied [47,48]. At the same time, in places like the United States, opioids are widely used among women, and are even more likely among women from lower socioeconomic strata [49]. We suggest that dentists should be trained in the use of low-level laser therapy and this technique should be preferably used alone to treat orofacial pain, or in combination with other therapies. In places like North America and Australia, where the indiscriminate use of opioid-related drugs has become an issue of public health proportions, the use of nonpharmacologic cost-effective therapies, such as low-level laser therapy, is safer for managing chronic pain than the long-term use of opioid analgesics [50].

The patient described here did not receive a diagnosis of TMD. Class II malocclusion may be associated with TMD but not necessarily with pain, and can be limited to crackling and bruxism [51]. The pain was a direct consequence of the surgical manipulation. In conclusion, dentists should be aware of low-level laser therapy as a treatment option, especially before prescribing opioids for long-term use. Further, we suggest that dentists should be trained in the use of low-level laser therapy and this technique should be preferably used to treat orofacial pain alone, or in combination with other therapies. This has the potential to improve quality of life or avoid loss of quality of life by the chronic use of opioids [52,53,54,55].

## Data Availability

All data are included in the manuscript.
